# Efeitos da Sexualidade na Funcionalidade Familiar e na Qualidade de Vida de Pessoas Idosas: Estudo Transversal**

**DOI:** 10.15649/cuidarte.2296

**Published:** 2022-08-28

**Authors:** Edison Vitório de, Benedito Fernandes da Silva Filho, Diego Pires Cruz, Randson Souza Rosa, Gabriel Magalhães Cairo, Cristiane dos Santos Silva, Lais Reis Siqueira, Namie Okino Sawada

**Affiliations:** 1 Universidade de São Paulo (EERP/ USP), Ribeirão Preto, São Paulo, Brasil. Email: edison.vitorio@usp.br Universidade de São Paulo Universidade de São Paulo Ribeirão Preto São Paulo Brazil edison.vitorio@usp.br; 2 Universidade Estadual do Sudoeste da Bahia (UESB), Jequié, Bahia, Brasil. Email: ditofilho13@gmail.com Universidade Estadual do Sudoeste da Bahia Universidade Estadual do Sudoeste da Bahia Jequié Brazil ditofilho13@gmail.com; 3 Universidade Estadual do Sudoeste da Bahia (UESB), Jequié, Bahia, Brasil. Email: diegopcruz@hotmail.com Universidade Estadual do Sudoeste da Bahia Universidade Estadual do Sudoeste da Bahia Jequié Bahia Brazil diegopcruz@hotmail.com; 4 Universidade Estadual do Sudoeste da Bahia (UESB), Jequié, Bahia, Brasil. Email: enfrandson@gmail.com Universidade Estadual do Sudoeste da Bahia Jequié Bahia Brasil enfrandson@gmail.com; 5 Universidade Estadual do Sudoeste da Bahia (UESB), Jequié, Bahia, Brasil. Email: leirbag@hotmail.com Universidade Estadual do Sudoeste da Bahia Universidade Estadual do Sudoeste da Bahia Jequié Bahia Brazil leirbag@hotmail.com; 6 Universidade Norte do Paraná (UNOPAR), Jequié, Bahia, Brasil. Email: cristianeimic@gmail.com Universidade Estadual do Norte do Paraná Universidade Norte do Paraná Jequié Bahia Brazil cristianeimic@gmail.com; 7 Universidade Federal de Alfenas (UNIFAL), Alfenas, Minas Gerais, Brasil. Email: laisreis.siqueira@gmail.com Universidade Federal de Alfenas Universidade Federal de Alfenas Alfenas Minas Gerais Brazil laisreis.siqueira@gmail.com; 8 Universidade Federal de Alfenas (UNIFAL), Alfenas, Minas Gerais, Brasil. Email: namie.sawada@unifal-mg.edu.br Universidade Federal de Alfenas Universidade Federal de Alfenas Alfenas Minas Gerais Brazil namie.sawada@unifal-mg.edu.br

**Keywords:** Saúde Pública, Saúde do Idoso, Assistência Integral à Saúde, Sexualidade, Relações Familiares, Public Health, Health of the Elderly, Comprehensive Health Care, Sexuality, Family Relations, Salud Pública, Salud del Anciano, Atención Integral de Salud, Sexualidad, Relaciones Familiares

## Abstract

**Introdução:**

a literatura já aponta que a família, muitas vezes, é o principal obstáculo que impede as pessoas idosas vivenciarem sua sexualidade. Porém, até onde sabemos, não existem estudos que identifiquem os efeitos da sexualidade na funcionalidade familiar.

**Objetivo:**

analisar os efeitos da sexualidade sobre a funcionalidade familiar e sobre a qualidade de vida de pessoas idosas.

**Materiais e métodos:**

estudo seccional realizado com 692 pessoas idosas entre os meses de julho e outubro de 2020 por meio da utilização de instrumentos autoaplicáveis. Os dados foram analisados com o teste *de Kruskal-Wallis,* correlação de *Pearson* e modelagem de equações estruturais.

**Resultados:**

as pessoas idosas com algum grau de disfuncionalidade familiar apresentaram pior vivência na sexualidade e pior qualidade de vida. O domínio relações afetivas da sexualidade foi o único a exercer efeito de forma positiva, de moderada a forte magnitude com a funcionalidade familiar (CP=0,472 [IC95%=0,301-0,642] p<0,001). A qualidade de vida, por sua vez, sofreu efeito positivo, de fraca a moderada magnitude, de todos os domínios da sexualidade: ato sexual (CP=0,339 [IC95%=0,190-0,488] p<0,001); relações afetivas (CP=0,117 [IC95%= -0,041-0,275] p<0,001) e adversidades física e social (CP=0,150 [IC95%=0,074-0,226] p<0,001).

**Conclusão:**

a sexualidade entre as pessoas idosas pode ser explorada com maior frequência nos serviços de saúde, uma vez que, exerceu efeitos positivos na funcionalidade familiar e na qualidade de vida dessa população. Espera-se que com os resultados deste estudo, haja valorização da temática nos serviços assistenciais e que a sexualidade na velhice seja explorada com as pessoas idosas, especialmente na atenção primária à saúde.

## Abstract (en)

**Introduction:**

the literature already points out that the family is often the main obstacle that prevents elderly people from experiencing their sexuality. However, there are no studies that identify the effects of sexuality on family functioning.

**Objective:**

analyzing the effects of sexuality on family functionality and on the quality of life of the elderly.

**Materials and methods:**

a cross-sectional study conducted with 692 elderly people between the months of July and October 2020 by self- administered instruments. Data were analyzed using the Kruskal-Wallis test, Pearson correlation and structural equation modeling.

**Results:**

the elderly with some degree of family dysfunction had a worse experience in sexuality and a worse quality of life. The affective relationships domain of sexuality was the only one to exert a positive effect, from moderate to strong magnitude on family functionality (PC=0.472 [CI95%=0.301-0.642] p<0.001). Quality of life, in turn, had a positive effect, from weak to moderate magnitude, in all domains of sexuality: sexual act (PC=0.339 [CI95%=0.190- 0.488] p<0.001); affective relationships (PC=0.117 [CI95%= -0.041-0.275] p<0.001) and physical and social adversities (PC=0.150 [CI95%=0.074-0.226] p<0.001).

**Conclusion:**

sexuality among the elderly can be explored more frequently in health services, as it exerted positive effects on family functionality and quality of life in this population. It is expected that with the results of this study, there is an appreciation of the theme in care services and that sexuality in old age is explored with the elderly, especially in primary health care.

## Resumen

**Introducción:**

la literatura ya señala que la familia es muchas veces el principal obstáculo que impide que las personas mayores experimenten su sexualidad. Sin embargo, hasta donde sabemos, no existen estudios que identifiquen los efectos de la sexualidad en el funcionamiento familiar.

**Objetivo:**

analizar los efectos de la sexualidad sobre la funcionalidad familiar y la calidad de vida de las personas mayores.

**Materiales y métodos:**

un estudio transversal realizado con 692 adultos mayores entre los meses de julio y octubre de 2020 utilizando instrumentos autoadministrados. Los datos se analizaron mediante la prueba de *Kruskal-Wallis*, la correlación de *Pearson* y el modelado de ecuaciones estructurales.

**Resultados:**

los ancianos con algún grado de disfunción familiar tuvieron peor experiencia en sexualidad y peor calidad de vida. El dominio de las relaciones afectivas de la sexualidad fue el único que ejerció un efecto positivo, de moderada a fuerte magnitud acerca de la funcionalidad familiar (CP = 0,472 [IC95% = 0,301-0,642] p <0,001). La calidad de vida, a su vez, tuvo un efecto positivo, de débil a moderada magnitud, en todos los dominios de la sexualidad: acto sexual (CP = 0,339 [IC95% = 0,190-0,488] p <0,001); relaciones afectivas (CP = 0,117 [IC95% =-0,041-0,275] p <0,001) y adversidades físicas y sociales (CP = 0,150 [IC95% = 0,074-0,226] p <0,001).

**Conclusión:**

la sexualidad entre los adultos mayores puede ser explorada con mayor frecuencia en los servicios de salud, ya que ejerció efectos positivos acerca de la funcionalidad familiar y la calidad de vida en esta población. Se espera que, con los resultados de este estudio, se aprecie la temática en los servicios de atención y que se explore la sexualidad en la vejez con los ancianos, especialmente en la atención primaria de salud.

## Introdução

Estima-se, mundialmente, que a população de idosos dobrará em 2050. No Brasil, conforme acompanhamento epidemiológico, o processo de envelhecimento está acontecendo com maior rapidez se comparado à Europa no início da transição demográfica. Esse processo também tem influência na família uma vez que há mudanças na constituição familiar, como o envelhecimento mútuo de todos os membros e demais fatores de desequilíbrio e desarmonia entre os seus integrantes^1^.

De acordo com a evolução social que vem acontecendo nos últimos anos, o conceito de família também tem sofrido modificações, o que fez surgir alguns componentes como o organizacional, estrutural, cultural e religioso, dificultando a sua compreensão. No entanto, sabe-se que a família exerce importante função na sociedade e, em especial, às pessoas idosas, notabilizando a função referente ao afeto, pertencimento, suporte e proteção. Trata-se de uma instituição complexa e singular em que as interações existentes devem ser exploradas pois as ações de apenas um membro têm poder para envolver todo o grupo^2^.

O funcionamento de uma família é definido pela forma em que os membros gerenciam rotinas e funções diárias, se comunicam e se relacionam emocionalmente uns com os outros^3^^-^^5^. Trata- se de um fenômeno complexo que especifica as características estruturais e organizacionais de um grupo familiar e a interação entre seus membros^5^. Nesta perspectiva, existem sistemas familiares funcionais (maduros) e disfuncionais (imaturos)^6^.

O sistema familiar funcional tem a capacidade de responder aos conflitos e eventos críticos com certo equilíbrio emocional; há resolutividade dos problemas sem desestruturação harmônica e sem sobrecarga entre os membros^6^. De modo geral, a família funcional é representada pela capacidade dos membros cumprirem e conciliarem suas funções de maneira clara e apropriada à identidade e vocação de seus integrantes, no tocante aos perigos e oportunidades que sobressaem na sociedade^7^. Já na família disfuncional, há priorização de interesses pessoais em detrimento do grupo e não há responsabilização de seus papéis dentro do sistema. Além do mais, as relações interpessoais são superficiais e instáveis, raramente há resolutividade das situações críticas, os membros não se adaptam conforme as situações e não há readequação de papéis quando necessário, o que provoca desarmonia no sistema familiar ^6^.

O convívio e o apoio familiar são fatores essenciais para a promoção de um envelhecimento ativo. Ressalta-se que a adaptação e convivência das pessoas idosas com suas famílias têm influência no seu desenvolvimento de modo geral^1^. Não obstante, o envelhecimento é um desafio^1^ e exige-se estratégias inovadoras de caráter natural que podem ser benéficas para a saúde psicossocial da pessoa idosa. Cita-se como exemplo a vivência saudável da sexualidade. Define-se sexualidade como um termo que reflete a multidimensionalidade da expressão individual quanto aos sentimentos, amor, toque, intimidade, carinho, companheirismo, abraço, afeto, inclusive o ato sexual propriamente dito. Observa-se que não podemos reduzir a sexualidade ao sexo, visto que se trata de um constructo mais amplo caracterizado por sentimentos, pensamentos e cognição^8^^-^^10^.

A literatura já aponta que a família, muitas vezes, é o principal obstáculo que impede as pessoas idosas vivenciarem sua sexualidade em nível nacional e internacional. Por exemplo, de acordo com estudos brasileiros^11^^-^^12^, a família contribui para o fortalecimento e reprodução de preconceitos sobre a sexualidade na velhice, culminando na supressão dos desejos pelas pessoas idosas e submissão ao sistema socio-familiar. Além do mais, outro estudo^13^ desenvolvido com pessoas idosas na Malásia identificou que a família ignora a realidade de que seu apoio atua como fator protetor contra a falta de intimidade na velhice, especialmente, no aspecto sexual.

Por fim, estudo de revisão^14^ desenvolvido por autora indiana revelou que a família começa a expressar atitudes estigmatizantes contra seus membros idosos, de modo que seus desejos e/ou anseios em sexualidade, sobretudo, o sexual, se tornam prejudicados. Do mesmo modo, desde 1999, Ribeiro^15^ já afirmava que “em família, os filhos são geralmente os primeiros a negar a sexualidade dos pais...”.

Nota-se por meio desses levantamentos que há diversos estudos realizados que consideram a relação entre a família e a sexualidade de seus membros idosos. Porém, todos eles consideram a família como variável independente, identificando o impacto da família nas vivências em sexualidade. No nosso estudo, a variável independente é a sexualidade, pois queremos investigar quais os efeitos dessas vivências na funcionalidade familiar, relação esta, escassa em meios científicos, até onde sabemos, por isso, justifica-se o desenvolvimento dessa investigação.

Estudos tem demostrado que a sexualidade entre as pessoas idosas constitui-se em uma necessidade humana básica^16^, tornando-se essencial para a manutenção da saúde^16^, bem estar ^17^ e qualidade de vida (QV)^18^^-^^20^. A QV envolve a percepção do indivíduo em relação a todos os aspectos que fazem parte de sua vida, ou seja, reflete a harmonia das realizações em diversas dimensões de sua rotina como a família, espiritualidade, lazer, atividade sexual, trabalho, dentre outras^21^. Trata-se de um termo subjetivo e multidimensional considerado como um indicador de saúde cujas potencialidades fortalecem e estimulam as ações assistenciais para a sua promoção^22^^-^^23^.

A Organização Mundial da Saúde (OMS) define a QV como “a percepção do indivíduo de sua posição na vida, no contexto da cultura e dos sistemas de valores nos quais ele vive e em relação aos seus objetivos, expectativas, padrões e preocupações”^24^. Ressalta-se que, esse referencial será adotado nesse estudo para sustentar a discussão sobre QV. Nossa hipótese é que a melhor vivência da sexualidade está associada a um sistema familiar funcional e a melhor percepção de QV entre as pessoas idosas, além de que a sexualidade exerce efeito forte e positivo sobre a funcionalidade familiar e sobre a QV dessa população. Se for confirmada significância estatística, esse estudo poderá servir como fundamento para começarmos a adotar novas estratégias de promoção e proteção à saúde da pessoa idosa com foco especialmente na sexualidade. Diante disso, o objetivo do presente estudo foi analisar os efeitos da sexualidade sobre a funcionalidade familiar e sobre a qualidade de vida de pessoas idosas.

## Métodos

Trata-se de um estudo analítico, descritivo, observacional e seccional realizado conforme as recomendações do checklist *Strengthening the Reporting of Observational Studies in Epidemiology* (STROBE). O estudo foi desenvolvido de forma *online* por meio da Rede Social *Facebook* entre os meses de julho e outubro de 2020.

A amostra foi calculada levando em consideração um erro amostral de 5%, nível de confiança de 95%, proporção conservadora de 50% e ajuste para população infinita, resultando em uma amostra mínima de 385 participantes. Entretanto, devido a possibilidade de perdas e insuficiência de preenchimento dos questionários, adicionou-se mais de 70% (n=307) ao cálculo, o que resultou em uma amostra final de 692 participantes. Conforme as estimativas do Facebook no período da coleta havia 3.200.000 (três milhões e duzentos mil) pessoas idosas elegíveis a participarem do estudo.

Foi considerado os seguintes critérios de inclusão: participantes com idade maior ou igual a 60 anos; de ambos os sexos; casados, em união estável ou com parceiro(a) fixa(a); residentes em comunidade de qualquer lugar do Brasil; com acesso à internet e com conta ativa na Rede Social *Facebook.* Foram excluídos do estudo todas as pessoas idosas hospitalizadas, residentes em instituições de longa permanência ou similares. Em virtude dos usuários serem ativos em redes sociais e possuírem habilidades com recursos tecnológicos que garantem acesso à essas redes (celular, laptop, computador e/ou tablet), dispensou-se a aplicação de instrumentos que avaliam o estado cognitivo.

Os pesquisadores criaram uma página no *Facebook* na qual foi publicada um convite para participação em que continha informações sobre a instituição de vínculo, contato dos pesquisadores responsáveis, critérios de inclusão e um *hyperlink* de acesso direto ao questionário, configurando-se como uma técnica de amostragem do tipo consecutiva não probabilística.

O questionário foi elaborado com a ferramenta *Google-Forms* e organizado em quatro inquéritos: biosociodemográfico, sexualidade, funcionalidade familiar e QV. Foram considerados elegíveis para a análise somente os instrumentos com 100% de preenchimento. Além disso, ressalta-se que nesse estudo todos os participantes responderam a todas as questões solicitadas.

Antes de terem acesso ao questionário, o participante era direcionado a uma página exclusiva para a Leitura do Termo de Consentimento Livre e Esclarecido (TCLE). Ao finalizarem a leitura, os participantes clicaram na opção “Aceito participar do estudo” disponível no rodapé do TCLE. Esse processo foi obrigatório e somente tiveram acesso aos instrumentos aqueles que aceitaram a participação.

O inquérito biosociodemográfico foi elaborado pelos pesquisadores e continha questões que permitiram traçar o perfil dos participantes como faixa etária, sexo, situação conjugal, religião, etnia, escolaridade, número de filhos, orientação sexual, orientação sobre sexualidade e localização geográfica.

O inquérito sexualidade continham as questões da Escala de Vivências Afetivas e Sexuais do Idoso (EVASI) construída e validada no Brasil em 2012^25^. A EVASI é uma escala psicométrica composta por 38 itens e três dimensões: ato sexual, relações afetivas e, adversidades física e social, cujas respostas são tipo *likert*, variando entre 1 (nunca) a 5 pontos (sempre)^25^. Não há ponto de corte para esse instrumento e sua análise é por meio de que, quanto maior o escore, melhor os participantes estão experienciando a sexualidade. A Escala EVASI possui confiabilidade satisfatória, atingindo um alfa de *Cronbach* de 0,96 para o ato sexual; 0,96 para as relações afetivas e, 0,71 para as adversidades física e social^25^.

O inquérito funcionalidade familiar foi elaborado por meio do instrumento APGAR de família validado para a população brasileira em 2001^26^. O acrômio APGAR refere-se a *Adaptation* (adaptação), *Partnership* (companheirismo), *Growth* (desenvolvimento), *Affection* (afetividade) e *Resolve* (capacidade resolutiva). Trata-se de um instrumento composto por cinco questões capaz de avaliar a satisfação dos participantes com o suporte familiar recebido. As questões são pontuadas em 2 pontos (sempre), 1 ponto (algumas vezes) e 0 ponto (nunca). O resultado final poderá ser interpretado da seguinte forma: família funcional (7 a 10 pontos), disfunção leve (4 a 6 pontos) e disfunção severa (0 a 3 pontos)^27^. O instrumento APGAR de família demonstrou boa consistência interna por meio do alfa de *Cronbach* no valor de 0,80^28^.

O inquérito QV foi estruturado com o instrumento *World Health Organization Quality of Life- Old* (WHOQOL-Old), validado e adaptado para a população brasileira. O WHOQOL-Old é específico para avaliar a QV da população idosa, é composto por 24 itens que estão distribuídos em seis facetas: habilidades sensoriais; autonomia; atividades passadas, presentes e futuras; participação social; morte e morrer e intimidade^29^. Esse instrumento não possui ponto de corte e os resultados são interpretados em escala ascendente em que a maior pontuação indica melhor percepção de QV e, consequentemente, menor pontuação indica pior QV. As respostas podem atingir uma pontuação total de 24 a 100 pontos e estão organizadas em escala do tipo *likert* (1 a 5)^30^. O WHOQOL-Old também demonstrou consistência interna satisfatória por meio dos coeficientes de *Cronbach* variando de 0,71 a 0,88^29^.

Vale ressaltar que, antes dos participantes começarem a responder aos inquéritos, exigiu-se o e-mail de forma obrigatória com o objetivo de evitar preenchimento múltiplo pelo mesmo participante e, com isso, reduzir as chances de vieses. Não obstante, os autores utilizaram mensalmente a estratégia de impulsionamento de postagem. Trata-se de uma opção disponível no *Facebook* que permitiu a ampliação da divulgação do convite para todo o território brasileiro, proporcionando incremento nas curtidas, compartilhamentos e engajamentos na postagem. Desta forma, conseguiu-se o alcance do tamanho amostral determinado.

Após constatar a não normalidade dos nossos dados^31^ pelo teste de *kolmogorov-Smirnov* (p<0,05), foi utilizada a estatística não paramétrica representada pelo teste de *Kruskal-Wallis,* por haver somente variáveis com mais de duas categorias. O nível de significância adotado foi de 95% (p<0,05) para todas as análises no *software* estatístico IBM SPSS® versão 25. As variáveis qualitativas foram apresentadas por meio de frequências absolutas e relativas e, as variáveis quantitativas, por meio de mediana e intervalo interquartílico (IQ).

Não obstante, testou-se a matriz de correlação para que se pudesse conhecer os caminhos (relações) a serem traçados na segunda etapa da análise, a modelagem de equações estruturais (SEM), realizada pelo *software* estatístico STATA versão 15. Procedeu-se então para a construção do modelo, composto por duas variáveis latentes: a qualidade de vida, formada pelos domínios estatisticamente significantes e, a funcionalidade familiar, constituída pelos domínios do APGAR familiar; e por três variáveis observáveis: dimensões da EVASI. Os resultados foram apresentados juntamente com seus coeficientes padronizados (CP) e intervalos de confiança 95% (IC95%), sendo interpretados de acordo com Kline (2012)^32^, onde um CP de 0,10 indica um efeito pequeno, de 0,30 um efeito médio e > 0,50 um efeito forte. Ressalta-se que, embora o estudo seja transversal, a análise por meio da SEM permite a detecção de efeitos de uma variável sob a outra.

Os seguintes índices de ajuste de modelo foram considerados: o *Comparative Fit Index* (CFI) e o *Tucker-Lewis index* (TLI), com valores mais próximos de 1 indicando melhor ajuste^33^; a *Standardized root mean square residual* (SRMR), com valor inferior a 0,08 considerado um bom ajuste e inferior a 0,10 aceitável^32^,(^34^; a *Root-Mean-Square Error of Approximation* (RMSEA), com seu intervalo de confiança de 90% (IC90%), cujos valores interpretados são: (0 = ajuste perfeito); (<0,05 = bom ajuste); (0,05-0,08 = ajuste moderado); (0,08-0,10 = ajuste medíocre) e (> 0,10 = ajuste inadequado)^35^; e o índice de ajuste absoluto *Adjusted Goodness-of-Fit Index* (AGFI) que varia entre 0 e 1 e é geralmente aceito que valores de 0,90 ou superiores indicam modelos bem ajustados^36^.

Considerando os aspectos éticos da Resolução 466/2012 e 510/2016 do Conselho Nacional de Saúde, o presente estudo foi aprovado pelo Comitê de Ética em Pesquisa da Escola de Enfermagem de Ribeirão Preto da Universidade de São Paulo no ano de 2020 sob Parecer nº 4.319.644. Além disso, os participantes receberam a segunda via do TCLE pelo e-mail, após leitura e conhecimento dos riscos, benefícios e relevância do estudo.

## Resultados

Dentreosparticipantes, observou-sepredominânciadepessoasidosasdosexomasculino(59,0%; n=408), com idade entre 60 e 64 anos (48,0%; n=332), católicos (54,3%; n=376), autodeclarados brancos (67,5%; n=467), com ensino superior (39,7%; n=275), casados (63,6%; n=440), que convivem com o cônjuge por tempo maior que 20 anos (62,4%; n=432), heterossexuais (87,0%; n=602), residentes na região sudeste (44,8%; n=310), que não moram com os filhos (67,2%; n=465) e que nunca receberam orientações sobre sexualidade pelos profissionais de saúde (78,8%; n=545). Além do mais, observou-se que a maioria das pessoas idosas convive em um sistema familiar funcional (60,5%; n=419), seguida de disfunção leve (30,5%; n=211) e disfunção severa (9,0%; n=62).

Observa-se na Tabela 1 que houve diferença estatisticamente significante entre as pessoas idosas católicas e espíritas em duas dimensões da sexualidade: ato sexual (p=0,023) e relações afetivas (p=0,020), além da QV (p=0,006), indicando desta forma, que as pessoas idosas espíritas melhor vivenciam sua sexualidade e possuem melhor QV quando comparadas com as católicas. Outro achado importante foi que as pessoas idosas com parceiro(a) fixo(a) melhor vivenciam sua sexualidade em todas as dimensões avaliadas quando comparadas com as casadas e em união estável, verificados pelo *post-hot de Bonferroni.*

O tempo de convivência foi outra variável que se associou estatisticamente a todas as dimensões da sexualidade, além da funcionalidade familiar e QV. Todavia, o *post-hot de Bonferroni* não evidenciou significância entre essas duas últimas variáveis analisadas. Os resultados indicam, principalmente, que as pessoas idosas que convivem com seus cônjuges por um período inferior a cinco anos diferem estatisticamente daquelas que possuem mais de 20 anos de convivência nas dimensões ato sexual (p<0,001) e relações afetivas (p<0,001).

Morar com os filhos diferiu estatisticamente das pessoas idosas que não possuem filhos. Essa diferença pode ser observada nas dimensões ato sexual (p=0,004) e relações afetivas (p=0,014), no qual nota-se que as pessoas idosas que não possuem filhos melhor vivenciam tais dimensões. Além do mais, as pessoas idosas que tem filhos, mas não moram juntos, possuem melhor funcionalidade familiar quando comparados com aquelas que não possuem filhos (p=0,032).

Receber orientações sobre sexualidade pelos profissionais de saúde se associou estatisticamente à melhor vivência das relações afetivas (p=0,0380 e melhor QV (p=0,001). Por fim, no que diz respeito à orientação sexual, os idosos homossexuais possuem melhor vivência no ato sexual (p=0,049), porém, a significância estatística não permaneceu após a aplicação do *pós-hoc* de *Bonferroni.* Além do mais, as pessoas idosas heterossexuais diferiram estatisticamente nas relações afetivas (p=0,002) e na QV (p=0,028), quando comparadas com os participantes de outras orientações sexuais.

**Tabela 1:** Análise das variáveis biosociodemográficas com a sexualidade, funcionalidade familiar e QV de pessoas idosas. Ribeirão Preto, São Paulo, Brasil, 2020 (n=692)

		Sexualidade			
Variáveis	Ato sexual	Relações afetivas	Adversidades física e social	Funcionalidade familiar	QV geral
			Postos médios		
Religião					
Católico	327,82†	326,99†	334,39	351,79	332,02†
Protestante	365,44	360,44	336,99	328,83	333,36‡
Espírita	405,69†	360,44	386,86	402,19†	426,69†
Origens africanas	414,81	394,96	315,00	317,69	309,04
Outras	340,38	358,04	337,26	321,65	348,80
Sem religião	343,71	338,04	394,65	299,08†	352,26‡
Valor p	0,023*	0,020*	0,099	0,027*	0,006*
Estado civil					
Casado	304,76†,‡	312,22†,‡	330,78†	357,83	333,30
União estável	393,05†	390,07†	362,26	342,55	371,57
Parceiro(a) fixo(a)	444,49‡	421,88‡	385,09†	311,62	367,62
Valor p	<0,001*	<0,001*	0,015*	0,063	0,071
Tempo de convivência					
≤ 5 anos	451,52†	432,33†	382,27	318,87	376,88
Entre 6 e 10 anos	413,44‡	399,31‡	409,68†	388,35	399,31
Entre 11 e 15 anos	340,24	339,77	312,19	280,38	354,41
Entre 16 e 20 anos	359,45	340,52	335,52	296,20	336,49
> 20 anos	308,18†,	317,29†,‡	332,13†	358,94	331,14
Valor p	<0,001*	<0,001*	0,009*	0,007*	0,046*
Mora com os filhos					
Sim	347,87†	334,91†	337,77	334,19	333,37
Não	337,75‡	344,07‡	346,85	357,12†	349,66
Não tenho filhos	452,33†,	439,32†,‡	388,36	274,72	375,32
Valor p	0,004*	0,014*	0,373	0,032*	0,430
Já teve orientação sobre sexualidade					
Sim	364,88	376,77	355,42	393,30	364,51
Não	341,54	338,33	344,09	333,88	341,64
Valor p	0,209	0,038*	0,539	0,218	0,001*
Orientação sexual					
Heterossexual	352,59	356,20†	351,92	352,70	354,67†
Homossexual	400,54	373,92	385,38	268,71	354,42
Bissexual	255,00	210,54	351,63	284,46	292,88
Outros	297,79	277,75†	289,02	315,37	280,32†
Valor p	0,049*	0,002*	0,092	0,151	0,028*

* Significância estatística pelo teste de *Kruskal-Wallis* (p<0,05)

†, ‡ Diferenças entre grupos pelo *post-hoc* de *Bonferroni*

De acordo com a Tabela 2, observa-se que as pessoas idosas com algum grau de disfuncionalidade familiar (leve ou severo), apresentaram as menores medianas em todas as dimensões da sexualidade e da QV, quando comparadas com as pessoas idosas pertencentes à família funcional. No que se refere à avaliação geral da sexualidade, nota-se que há melhor vivência das relações afetivas [75,00 (65,00-81,00)]. Já para a QV, as habilidades sensoriais apresentaram a maior mediana [81,25 (68,75-93,75)], indicando que as pessoas idosas possuem melhor QV nesta faceta.

**Tabela 2:** Sexualidade e QV dos idosos conforme classificação da funcionalidade familiar. Ribeirão Preto, São Paulo, Brasil, 2020 (n=692)

	Funcionalidade Familiar				
	Disfunção Severa	Disfunção Leve	Funcional	valor de p	Avaliação Geral
	Mediana(I Q)	Mediana(I Q)	Mediana(I Q)		Mediana (I Q)
Sexualidade					
AS	63,00	72,00	76,00	<0,001*	74,00
	(50,75-78,00)	(60,00-80,00)	(68,00-81,00)		(64,00-80,00)
RA	62,00	72,00	77,00	<0,001*	75,00
	(44,75-78,25)	(59,00-80,00)	(69,00-82,00)		(65,00-81,00)
AFS	10,00	10,00	11,00	<0,001*	11,00
	(7,00-12,00)	(9,00-12,00)	(9,00-13,00)		(9,00-13,00)
Qualidade de vida					
HS	78,12	75,00	81,25	<0,008*	81,25
	(67,18-93,75)	(62,50-93,75)	(68,75-93,75)		(68,75-93,75)
AUT	50,00	62,50	75,00	0,001*	68,75
	(37,50-68,75)	(50,00-75,00)	(56,25-81,25)		(56,25-75,00)
APPF	50,00	62,50	75,00	0,001*	68,75
	(37,50-64,06)	(50,00-75,00)	(62,50-81,25)		(56,25-81,25)
PS	50,00	62,50	75,00	0,001*	68,75
	(35,93-68,75)	(50,00-75,00)	(62,50-81,25)		(56,25-75,00)
MM	68,75	68,75	75,00	0,025*	75,00
	(43,75-93,75)	(43,75-87,50)	(50,00-87,50)		(50,00-87,50)
INT	53,12	68,75	75,00	<0,001*	75,00
	(31,25-70,31)	(56,25-75,00)	(68,75-87,50)		(62,50-81,25)
QVG	57,81	64,58	75,91	<0,001*	68,75
	(46,87-68,75)	(56,25-72,91)	(64,58-81,25)		(59,63-79,16)

*Significância estatística para o teste de *Kruskal-Wallis* (p<0,05)

AS: ato sexual; RA: relações afetivas; AFS: adversidades física e social; HS: habilidades sensoriais; AUT: autonomia; APPF: atividades passadas, presentes e futuras; PS: participação social; MM: morte e morrer; INT: intimidade; QVG: QV geral

As correlações entre a QV, funcionalidade familiar e a sexualidade mostram-se positivas e significantes em sua totalidade, a exceção da relação das adversidades física e social com a funcionalidade familiar e intimidade, conforme observado na Tabela 3.

**Tabela 3:** Coeficiente de correlação de Pearson (r) entre sexualidade, QV e funcionalidade familiar. Ribeirão Preto, São Paulo, Brasil, 2020 (n=692)

			Sexualidade			
	Ato sexual		Relações afetivas		Adversidades física e social	
	r	p	r	p	r	p
DOM 2	0,411	<0,001	0,424	<0,001	0,102	<0,001
DOM 3	0,378	<0,001	0,384	<0,001	0,166	<0,001
DOM 4	0,317	<0,001	0,297	<0,001	0,151	<0,001
DOM 6	0,582	<0,001	0,631	<0,001	0,149	>0,05
APGAR	0,334	<0,001	0,409	<0,001	0,06	>0,05

DOM 2 - Autonomia; DOM 3 - Atividades passadas, presentes e futuras; DOM 4 - Participação Social; DOM 6 - Intimidade

**Figura 1:**
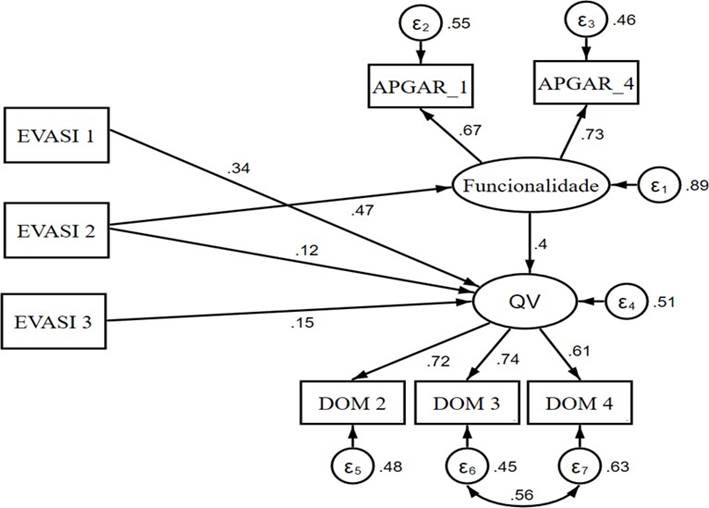
Modelo de equação estrutural para sexualidade, QV e funcionalidade familiar. Ribeirão Preto, São Paulo, Brasil, 2020

A análise dos componentes de mensuração do modelo permitiu constatar que, para a latente QV, apenas os domínios autonomia (DOM 2), atividades passadas, presentes e futuras (DOM 3) e participação social (DOM 4), apresentaram carga fatorial satisfatória para ser mantida no modelo. Já para a funcionalidade familiar, apenas as observáveis APGAR_1 e APGAR_4. O APGAR_1 se refere ao quanto os idosos estão satisfeitos em poder recorrer à família em busca de ajuda quando algo os incomoda ou os preocupa. O APGAR_4 se refere ao quanto as pessoas idosas estão satisfeitas com a maneira em que a família demonstra afeição e reage às suas emoções como mágoa, amor e raiva. Essas variáveis, juntamente com as três dimensões da sexualidade (EVASI), compuseram o modelo de mensuração aqui proposto, conforme Figura 1. Nota-se a adequação da totalidade dos índices de ajustamento RMSEA [0,045 (IC95% 0,02- 0,06)], CFI (0,987) e SRMR (0,02).

Quanto aos efeitos, nota-se na Tabela 4 que, o domínio “relações afetivas” (EVASI 2) foi o único a relacionar-se de forma positiva, de moderada a forte magnitude com a funcionalidade familiar. A QV, por sua vez, sofreu efeito positivo, de fraca a moderada magnitude, de todos os domínios da sexualidade, bem como da funcionalidade familiar.

**Tabela 4:** Coeficientes padronizados (CP) da modelagem por equações estruturais entre Funcionalidade Familiar, Sexualidade e QV. Ribeirão Preto, São Paulo, Brasil, 2020 (n=692)

	CP	IC95%		P
Modelo de mensuração				
APGAR 1 ← Funcionalidade	0,669	0,582	- 0.754	<0,001
APGAR 4 ← Funcionalidade	0,715	0,644	- 0,822	<0,001
DOM 2 ← QV	0,722	0,663	- 0,781	<0,001
DOM 3 ← QV	0,742	0,682	- 0,801	<0,001
DOM 4 ← QV	0,611	0,543	- 0,680	<0,001
Modelo Estrutural				
Funcionalidade ← EVASI 2	0,472	0,301	- 0,642	<0,001
QV ← Funcionalidade	0,403	0,306	- 0,500	<0,001
QV ← EVASI 1	0,339	0,190	- 0,488	<0,001
QV ← EVASI 2	0,117	-0,041	- 0,275	<0,001
QV ← EVASI 3	0,150	0,074	- 0,226	<0,001

DOM 2 - Autonomia; DOM 3 - Atividades passadas, presentes e futuras; DOM 4 - Participação Social; DOM 6 - Inti- midade; EVASI 1 - Ato sexual; EVASI 2 - Relações Afetivas; EVASI 3 - Adversidades física e social

## Discussão

A maioria dos participantes desse estudo convivem em um sistema familiar funcional (60,5%; n=419), seguida de disfunção leve (30,5%; n=211) e disfunção severa (9,0%; n=62), corroborando com outros estudos brasileiros realizados com pessoas idosas^1^ e com alguns cuidadores de pessoas idosas maiores de 60 anos^37^ que também identificaram essa mesma proporcionalidade no que diz respeito à funcionalidade familiar avaliada com o mesmo instrumento.

Observou-se também, que as pessoas idosas espíritas melhor vivenciam sua sexualidade nas dimensões ato sexual e nas relações afetivas, além de possuírem melhor QV quando comparadas com as pessoas idosas católicas. Esses resultados podem ser justificados, em partes, pelo conservadorismo em relação à sexualidade predominar no catolicismo. Destaca- se que, a religião é considerada uma das principais barreiras que dificultam as vivências da sexualidade, especialmente na velhice, em que o ato sexual, por exemplo, é tido como algo impuro e indigno^38^. Corroborando com essa inferência, um estudo^39^ brasileiro desenvolvido com 241 pessoas idosas no Estado de Pernambuco revelou que, aquelas adeptas ao catolicismo e protestantismo, demonstraram atitudes mais conservadoras no que concerne a sexualidade na velhice.

Outro achado importante foi que as pessoas idosas com parceiro(a) fixo(a) melhor vivenciam sua sexualidade em todas as dimensões avaliadas. Nesse estudo, as pessoas idosas com parceiro fixo são aqueles que não são casadas e nem estão em união estável, mas mantém relacionamentos íntimos com uma pessoa específica. Nesse sentido, esperava-se que, as pessoas idosas casadas tivessem as melhores pontuações na avaliação da sexualidade, pois o casamento é idealizado, principalmente no Brasil, como em um espaço em que há maior liberdade de expressão íntima^40^. Essa liberdade não é observada nas pessoas idosas que não estão dentro do matrimônio, pois a carga social de preconceitos que já incidem sobre elas e, as inibem de qualquer expressão em sexualidade^41^, se fortalece ainda mais quando as pessoas idosas não estão inseridas no espaço do casamento.

O tempo em que as pessoas idosas convivem com os cônjuges também apresentou diferença estatisticamente significante. Os participantes que convivem por um período inferior a cinco anos possuem melhor vivência nas dimensões do ato sexual e nas relações afetivas quando comparados com aqueles que possuem mais de 20 anos de convivência. Esse achado pode ser explicado em virtude do longo tempo que os cônjuges permanecem juntos, podendo evoluir para um certo estado de comodismo decorrente da rotina e monotonia do dia-a-dia^42^, fato que não é observado, por exemplo, entre os indivíduos com pouco tempo de convivência com seus parceiros.

Encontrou-se no presente estudo que as pessoas idosas que não possuem filhos melhor vivenciam o ato sexual e as relações afetivas. De certa forma, esse resultado corrobora com uma investigação^43^ transversal realizada com 200 pessoas idosas brasileiras do Estado do Pará, na qual houve relatos de que a família se constitui um fator impeditivo para as vivências da sexualidade na velhice. Outro estudo^11^ brasileiro com abordagem qualitativa realizado com mulheres idosas no estado do Paraná identificou que, dentre outras, a opressão familiar dificulta a vivência plena da sexualidade pelas participantes. Esses estudos sustentam os resultados encontrados na presente investigação de que a ausência de filhos corrobora para que eles expressem melhor suas vivências em sexualidade.

Outro achado de extrema relevância para as práticas assistenciais em saúde, diz respeito ao fato de que, as pessoas idosas que receberam orientações sobre sexualidade pelos profissionais de saúde evidenciaram melhor vivência nas relações afetivas e melhor QV. Entretanto, embora seja reconhecida cientificamente os benefícios da sexualidade para a saúde, bem-estar e QV^44^, existem obstáculos que precisam ser superados, especialmente, na relação profissional- paciente.

Isto porque alguns estudos revelam que, por um lado, as pessoas idosas sentem medo, receio e/ ou vergonha de perguntar ao profissional de saúde sobre aspectos de sua sexualidade^45^^-^^46^ e, por outro lado, os profissionais não questionam a seus pacientes sobre a temática, seja por falta de capacitação e/ou deficiência durante a formação profissional^47^^-^^48^. Talvez, essas evidências possam justificar, inclusive, a alta taxa (78,8%) de pessoas idosas que nunca receberam orientações sobre sexualidade pelos profissionais de saúde no presente estudo. Como consequência, as pessoas idosas assumem, de certa forma, uma posição de desvantagem por não desfrutarem dos prazeres e benefícios que a sexualidade proporciona.

Essa alta taxa pode estar relacionada à detenção de atitudes conservadoras decorrente da deficiência no processo de formação além da influência dos valores morais e sociais presentes. Nesse sentido, um estudo^48^ brasileiro desenvolvido com enfermeiros da Estratégia de Saúde da Família identificou que a maioria desses profissionais possui conhecimento acerca da sexualidade na velhice, porém, possuem atitudes conservadoras em relação à temática. Além disso, 94,6% dos profissionais afirmaram saber orientar a pessoa idosa em questões relacionadas à sexualidade, porém, 75% deles não realizam atividades educativas sobre a temática com esse público^48^. Desse modo, os autores ressaltam a imprescindibilidade de implementação de estratégias educativas permanentes com foco na ampliação do conhecimento dos profissionais e, consequentemente, aperfeiçoamento das práticas assistenciais^48^.

Isto porque de acordo com outro estudo^49^ brasileiro desenvolvido com 477 pessoas idosas, observou-se que os participantes que alguma vez receberam orientações sobre sexualidade por algum profissional da saúde, melhor vivenciaram sua sexualidade tanto nos aspectos sexuais quanto nos afetivos, além de melhor encararem os obstáculos sociais para a sua vivência, o que reforça a evidência de que capacitar os profissionais de saúde é a melhor estratégia a ser feita. Ainda nessa perspectiva, precisa-se, também, reorientar os processos de formação em enfermagem quanto a integralidade assistencial à saúde da pessoa idosa, fortalecendo a articulação entre a teoria e prática, especialmente no que diz respeito à sexualidade na velhice, que deve dialogar com os aspectos socioculturais para que se alcance uma assistência holística, resolutiva^48^, e isenta de preconceitos e julgamentos^50^, afinal, a sexualidade na velhice é natural, prazerosa e saudável gerando, portanto, bem estar aos envolvidos^38^.

Nesse estudo, o modelo por equações estruturais indicou que, a dimensão relações afetivas foi a única a relacionar-se de forma positiva, de moderada a forte magnitude com a funcionalidade familiar. Esse resultado indica que, quanto mais as pessoas idosas se aprofundam em suas relações afetivas referentes à sua sexualidade, melhor será a funcionalidade familiar, demonstrando efeito positivo entre essas duas variáveis.

Todavia, a literatura evidencia barreiras entre as famílias que dificultam as vivências da sexualidade pelas pessoas idosas. Nesse contexto, embora a família assuma papel de destaque no incentivo e apoio na velhice, quando se trata da sexualidade, há intensificação de preconceitos que culmina na ridicularização e supressão da sexualidade na terceira idade^12^. Em decorrência disso, as pessoas idosas ficam expostas à estressores que podem influenciar negativamente em sua saúde, pois a supressão da sexualidade pode acelerar o processo de envelhecimento e causar impactos indesejáveis na sua saúde^51^.

A QV, por sua vez, sofreu efeito positivo, de fraca a moderada magnitude, de todos os domínios da sexualidade. Isso significa que, as vivências da sexualidade impactam beneficamente à QV desse grupo etário e, por isso, torna-se necessário que os profissionais de saúde, especialmente na atenção primária, conduzam adequadamente as consultas em saúde de forma holística, garantindo sobretudo, os aspectos da sexualidade da pessoa idosa.

A atenção primária é uma das portas de entrada aos serviços de saúde e caracteriza-se pela longitudinalidade e coordenação do cuidado, tendo as práticas educativas como uma das tecnologias de cuidado frequentemente adotada, seja por meio de grupos ou consultas individuais^52^. A educação em saúde voltada para a sexualidade proporciona o empoderamento social com importantes contribuições para a QV e para uma visão positiva da sexualidade no envelhecimento. Existem atualmente diversas metodologias ativas que podem ser aplicadas na atenção primária durante as práticas educativas em sexualidade na velhice. Tais metodologias são conhecidas por superar o modelo tradicional que estabelece relação de passividade ao indivíduo, tornando-se, portanto, uma tecnologia crítica, reflexiva e participativa que coloca os educandos como agentes centrais de seu apredizado^52^.

Nesse sentido, um estudo^53^ de pesquisa ação-educativa desenvolvido com mulheres idosas identificou que essa abordagem constituiu-se uma importante ferramenta do cuidado, pois permitiu fragilizar os preconceitos existentes sobre a temática, promoveu saúde das participantes e evidenciou novas alternativas para o cuidar. Além disso, as autoras reforçam que essa metodologia apresenta aplicabilidade na atenção primária à saúde no intuito de fornecer um cuidado emancipador em saúde^53^.

A literatura ratifica a relação direta que existe entre a sexualidade e QV^46^, além da função relevante ao longo dos anos vividos mediante novas formas de obtenção do prazer, autoconhecimento,

autoestima e bem-estar^38^. Inclusive, as próprias pessoas idosas referem que a sexualidade se constitui como um aspecto fundamental para sua QV^46^. Assim, os profissionais de saúde devem considerar que a sexualidade da pessoa idosa deve, dentre outras coisas, promover melhor QV e saúde, minimizando as negligências assistenciais que, predominantemente, faz parte da assistência a esse público^54^.

Porém, vale destacar que, a limitação quantitativa de estudos relacionados à sexualidade da pessoa idosa se caracteriza como um dos principais desafios para os pesquisadores e profissionais de saúde. Há escassez de pesquisas que abordam a sexualidade em seu significado holístico, prevalecendo as investigações com enfoque nas disfunções sexuais e no declínio fisiológico decorrente do processo de envelhecimento^55^.

## Limitações do estudo

Esse estudo apresenta algumas limitações que devem ser consideradas. Primeiramente, o delineamento não probabilístico fragiliza a validade externa dos resultados. Além disso, em virtude da coleta de dados ser *online*, consequentemente, houve seleção restrita de participantes com maior nível socioeconômico, o que pode ser confirmado, por exemplo, pela alta prevalência de idosos com ensino superior, realidade pouco frequente entre a maioria das pessoas idosas brasileiras.

## Conclusão

O presente estudo permitiu concluir que a dimensão relações afetivas da sexualidade foi a única a relacionar-se de forma positiva, de moderada a forte magnitude com a funcionalidade familiar. A qualidade de vida, por sua vez, sofreu efeito positivo, de fraca a moderada magnitude de todos os domínios da sexualidade. Logo, constata-se que a sexualidade entre as pessoas idosas pode ser explorada com maior frequência nos serviços de saúde, uma vez que, exerceu efeitos positivos na funcionalidade familiar e na qualidade de vida dessa população. Assim, espera-se que com os resultados deste estudo, haja valorização da temática nos serviços assistenciais e que a sexualidade na velhice seja explorada com as pessoas idosas, especialmente na atenção primária à saúde, em que os enfermeiros poderão investir na criação de agenda específica na ESF, que contemple o ser idoso em todas as suas dimensões da vida, em particular, no que condiz com sua sexualidade.

Por fim, sugerimos ainda, que a sexualidade entre as pessoas idosas seja debatida com mais profundidade durante a formação profissional em saúde no intuito de romper com o elo de preconceitos que dificulta o diálogo sobre a temática entre o profissional e paciente. Desse modo, o profissional terá maior confiança e habilidades capazes de conduzir a discussão sobre sexualidade com seus pacientes como forma de promoção e proteção à saúde e à QV.

Nesse sentido, acreditamos que a educação no campo do envelhecimento sustenta o compromisso com a velhice ativa e colabora para o rompimento de preconceitos e crenças errôneas que reduzem à velhice a um estágio terminal e incapaz de oferecer prazer. Destacamos, então, que a educação transversaliza os ambientes de formação e atuação profissional, podendo ser explorada por meio de diversas metodologias individuais e/ou grupais e que por meio dela, poderemos construir uma sociedade mais justa e igualitária em diversas áreas de interesse social como gênero, sexualidade, envelhecimento e minorias populacionais.
